# Severe congenital neutropenia due to G6PC3 deficiency: early and delayed phenotype in two patients with two novel mutations

**DOI:** 10.1186/s13052-014-0080-8

**Published:** 2014-11-14

**Authors:** Lucia Dora Notarangelo, Gianfranco Savoldi, Sara Cavagnini, Veronica Bennato, Sabrina Vasile, Alba Pilotta, Alessandro Plebani, Fulvio Porta

**Affiliations:** Pediatric Onco-haematology and BMT Unit, Children’s Hospital, Spedali Civili of Brescia, Brescia, Italy; Department of Pathology, Laboratory of Genetic Disorders of Childhood, A. Nocivelli Institute for Molecular Medicine, Spedali Civili, Brescia, Italy; U.O. di Pediatria, Ospedale Valduce, Como, Italy; Pediatrics Clinic, Spedali Civili of Brescia, Brescia, Italy; Department of Clinical and Experimental Science, Pediatrics Clinic and Institute of Molecular Medicine “A. Nocivelli”, University of Brescia and Spedali Civili of Brescia, Brescia, Italy

**Keywords:** Neutropenia, Severe congenital neutropenia type 4, SCN4, G6PC3, G-CSF

## Abstract

Severe Congenital Neutropenia type 4 (SCN4, OMIM 612541) is a rare autosomal recessive disease due to mutations in the *G6PC3* gene. The phenotype comprises neutropenia of variable severity and other anomalies including congenital heart defects, prominent superficial veins, uro-genital anomalies, facial dysmorphism, growth and developmental delay and intermittent thrombocytopenia. In some patients, SCN represents the only manifestation of the disease. Variable findings have been reported at bone marrow examination ranging from a maturation arrest at the myelocyte/promyelocyte stage (either in a hypocellular or hypercellular context) to myelokathexis. Here we report two patients harbouring two novel mutations in the *G6PC3* gene, including the first Italian patient even described. Both the patients share profound neutropenia with severe infections early in life; in one case non-hematopoietic stigmata of the syndrome, including evident facial dysmorphism and vascular anomalies, appeared gradually over time, prominently in the second decade. Therefore, G6PC3 defects should be considered in any case of congenital, unexplained neutropenia regardless of the clinical phenotype. Both patients are on G-CSF treatment with no evidence of malignant evolution. Even if G6PC3 deficiency seems not to have a propensity towards malignancy, a careful evaluation is warranted.

## Background

Severe congenital neutropenia (SCN) is a heterogeneous group of mature neutrophil deficiency disorders with or without immunological or extra-hematopoietic abnormalities [1]. Various genetic defects can cause SCN, and some of them also predispose to MDS/AML [2]. SCN type 4 (SCN4, OMIM 612541) is a rare autosomal recessive disease due to mutations in the *G6PC3* gene which encodes for the catalytic subunit of the glucose-6-phosphatase 3 protein (G6Pase3) [3]. Along with G-6Pase, which is mutated in Glycogen Storage Disease Type 1 (GSD1), G-6Pase 3 belongs to the glucose-6-phosphatase (Glc-6-Pase) family, a group of transmembrane, endoplasmic reticulum (ER)- associated proteins [3]. Although the two isoenzymes share the same catalytic properties, only defects in G-6Pase are associated with metabolic symptoms, reflecting their unique role in fasting glucose homeostasis and different tissue expression patterns [3].

The human *G6PC3* gene is located at chromosome 17q21 and has 6 exons. SNC4 is most often due to missense mutations. Moreover, some mutations occur with higher frequency in certain ethnic groups, possibly reflecting a founder effect [4,5]. Since the first description, 59 patients with SCN4 have been reported. All of them share neutropenia of variable severity and some have other anomalies including congenital heart defects, prominent superficial veins, uro-genital anomalies, facial dysmorphism, growth and developmental delay and intermittent thrombocytopenia [4,6]. In some patients, SCN represents the only manifestation of the disease [7]. Mutation of the same gene may give rise to Dursun Sindrome (DS) (OMIM 613034), a more severe, often lethal, phenotype [8]. This syndrome is characterized by a triad of familial primary pulmonary hypertension (PPH), leucopenia and atrial septal defects [9]. Other features include secundum-type atrial septal defect, intermittent neutropenia, lymphopenia, monocytosis, anemia, and thymic hypoplasia. The prognosis is often poor due to severe respiratory distress [9]. Here we report two patients harbouring two novel mutations in *G6PC3* gene, including the first Italian patient who is also, to our knowledge, the longest survivor with SCN4 on treatment with G-CSF ever described.

## Case report

### Patient 1

**Figure 1:**
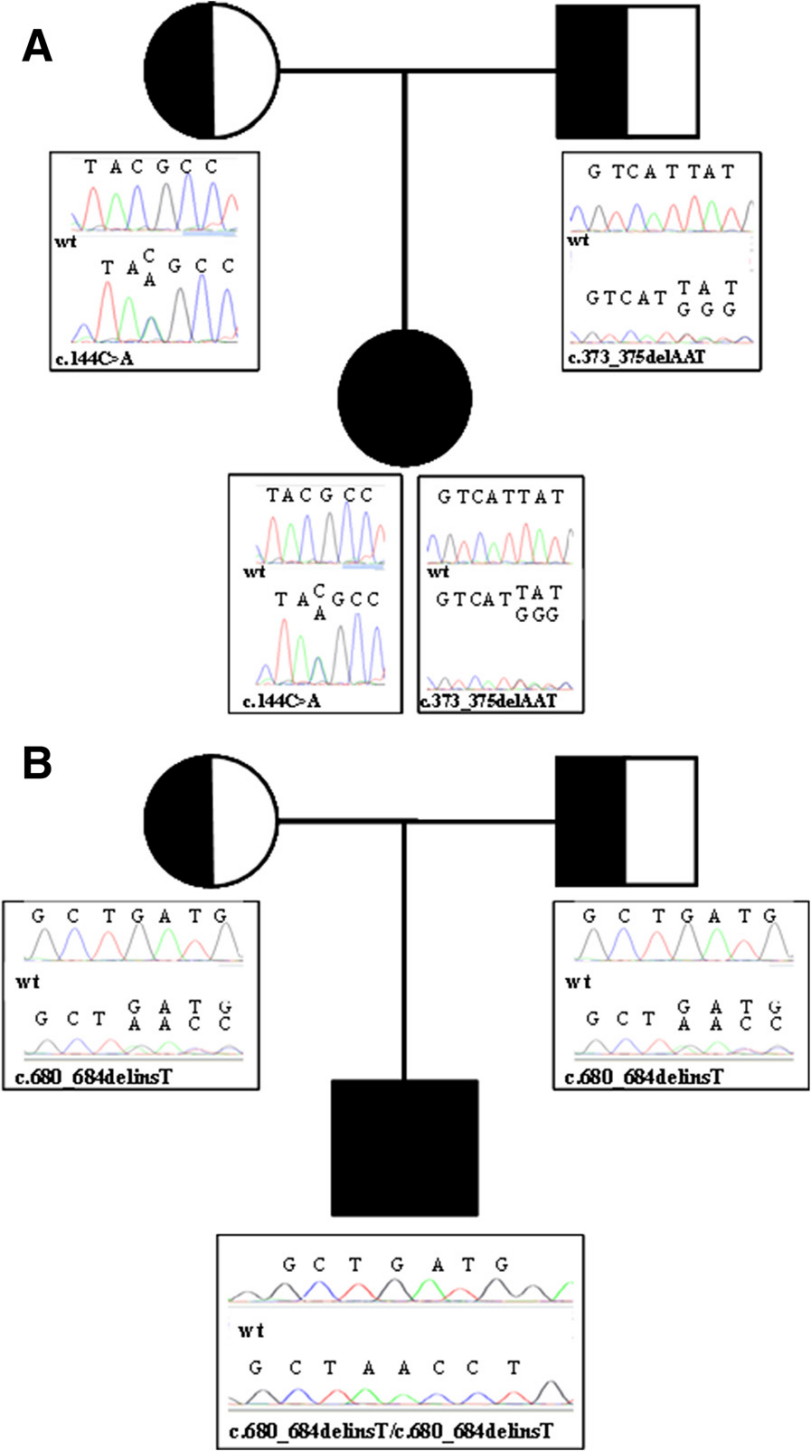
**Segregation analysis of the mutations identified in patient 1 (A) and patient 2 (B).**

The proband is a 20 year old female, second child of healthy and unrelated Italian parents, born at term after an uneventful pregnancy. She developed recurrent and serious *S. aureus* infections since early in life (skin infection at 10 days of age, pulmonary abscess at 2 months, brain abscess leading to lateralized seizures at 5 months) (Table 1). Complete blood cell count showed normal white blood cell count (6.600/μL) with severe neutropenia (220 cells/μL), monocytosis (3,400 cells/μL), mild anemia (Hb 8.6 g/dl, MCV 78 fl) and thrombocytosis (610 × 109/L). Immunological evaluation, including serum immunoglobulins, lymphocyte subsets, expression of adhesion molecules, complement, dihydrorhodamine (DHR) test, was normal (Table 1). Due to persistent neutropenia, a bone marrow aspiration was performed, which showed global hypercellularity with myeloid hyperplasia, maturation arrest at the promyelocyte-myelocyte stage and paucity of mature neutrophils. Treatment with G-CSF was started (5–10 μg/Kg/d) with normalization of ANC (>1,500 cell/μl). At 8 yrs of age, the patient developed thrombocytopenia (33 × 10^9^/L), which was initially attributed to G-CSF treatment. Subsequently the platelet count spontaneously increased with an intermittent course (Table 1). Reduced height growth speed was observed since 10 years of age. Because of delayed puberty, a decapeptyl test was performed at 16 years, and results were consistent with hypergonadotrophic hypogonadism (data not shown). A facial dysmorphism with frontal bossing, upturned nose and retrognatia became more evident over time, prominently in the second decade, along with the appearance of edema and superficial venous pattern of the lower limbs around 13 years. Other non hematological aspects are reported in Table 1. Upon informed consent, the coding region of the *G6PC3* gene was sequenced, revealing compound heterozygosity for c.144C > A substitution (predicted to result in p.Y48* premature termination) and a novel three nucleotide deletion (c.373_375delAAT), predicted to cause deletion of the Isoleucine residue at position 125 (p.I125del). Each parent was found to be heterozygous for one of the two mutations (see Figure 1A). In order to define the pathogenic role of the variants identified in our patients, we performed DHPLC analysis on 150 samples of genomic DNA (300 chromosomes) from unrelated healthy control individuals [10]. Analysis by DHPLC of the PCR fragments containing the mutations did not show any alterations of the DHPLC profiles in the control subjects analysed. The patient is currently 20 years old without major infections or developmental delay, on G-CSF treatment (2 μg/Kg/d). Yearly bone marrow evaluations have confirmed previous findings.

**Table 1:** **Hematological and immunological evaluation, phenotype and mutations in G6PC3 patients**

		**Patient**	
		**1**	**2**
	**Eth**	**Italian**	**Turkish (consanguinous)**
	**Sex**	**F**	**M**
	**Current age (years)**	**20**	**3**
	**Infections prior to G-CSF therapy**	**Skin, pulmonary and brain abscess by** ***S. Aureus***	**Otitis, parotitis,** ***S. Viridans*** **sepsis,** ***S. Aureus*** **gluteal abscess, recurrent aphtous stomatitis**
**Blood cells count**	**White blood cells ^ (4.5-17)**	**6,6**	**2,7**
	**Neutrophils ^ (1.5-6.0 × 109/L)**	**0,22**	**0,06**
	**Lymphocytes ^ (1.3-8.5 × 109/L)**	**2,9**	**1,9**
	**Monocytes ^ (0.1-1.0 × 109/L**	**3,4**	**0,7**
	**Platelet count ^ (150–450 cells/uL)**	**610**	**347**
	**Intermittent thrombocytopenia**	**Yes (range 46–158 cells/uL)**	**No**
**Serum Immunoglobulin concentrations**	**IgG (mg/dL)**	**1.240 (231–947)**	**974 (462–1710)**
	**IgA (mg/dl)**	**54 (8–74)**	**64 (27–173)**
	**IgM (mg/dl)**	**79 (26–210)**	**118 (62–257)**
	**IgE (kU/L) (n.v. < 95)**	**3**	**7**
**Lymphocyte subset (cells/uL)**	**CD3+ (770–1880)**	**720**	**1287**
	**CD3 + CD4+ ( 470–1240)**	**373**	**805**
	**CD3 + CD8+ (215–730)**	**318**	**426**
	**CD19+ (100–390)**	**218**	**460**
	**CD16+ (70–550)**	**69**	**79**
	**CD11b (PMN)**	**98%**	**N.D.**
	**CD18+ (PMN)**	**100%**	**N.D**
	**Extra haematological features**	**Facial dysmorphisms,**	**Facial dysmorphisms,**
		**mitral valve prolapse,**	**prominent veins,**
		**prominent veins,**	**sensorineural hearing loss,**
		**ligamentous laxity,**	**micropenis, coronal ipospadia.**
		**inguinal hernia,**	
		**delayed puberty,**	
		**hypergonadotrophic hypogonadism**	
	**G6PC3**	**c.144C>A/c.373_375delAAT**	**c.680_684delinsT/ c.680_684delinsT**
	**Mutation**	p.Y48*/ p.I125del	p.S227Lfs*3/ p.S227Lfs*3

^ prior to G-CSF therapy.

NA: not available.

Immunoglobulins normal value for age are reported in brackets.

### Patient 2

A male patient, third child of consanguineous Turkish parents, was referred to us at the age of 2 years for severe neutropenia and early-onset recurrent infections including otitis, parotitis, S. viridans sepsis, S. aureus gluteal abscess, and recurrent aphtous stomatitis (Table 1). Mild sensorineural hearing loss was diagnosed at 4 months of life. At 6 months of age, the patient developed chickenpox without complications. Bone marrow aspiration, performed at another institution, revealed delayed granulocyte maturation. No mutations were found in the *ELANE* and *HAX1* genes. On physical examination, the weight was 13 Kg (25-50^th^ percentile), and length was 88 cm (50^th^ percentile). There was facial dysmorphism with frontal bossing, upturned nose and malar hypoplasia, prominent superficial venous pattern on the arms, legs and trunk, and micropenis with coronal ipospadia. Laboratory investigations revealed severe neutropenia (ANC: 140 cells/μL) with normal lymphocyte, monocyte and platelet counts, slight microcytic anemia (Hb 10,5 g/dl; MCV 65,4 fl), normal serum immunoglobulins, protective antibody response to tetanus toxoid, and normal distribution of lymphocyte subsets. Abdominal ultrasound and echocardiography were also normal (see Table 1). Upon informed consent, mutation analysis of the *G6PC3* gene was performed and revealed a novel homozygous 5 bp indel mutation (c.680_684delinsT) in exon 6, resulting in Serine to Leucine substitution at position 227, and frameshift with premature termination (p.S227Lfs*3). Both parents were found heterozygous for the mutation (see Figure 1B). DHPLC analysis on 150 samples of genomic DNA (300 chromosomes) from unrelated healthy control individuals didn’t show any alterations [10]. Treatment with G-CSF (5 μg/Kg/d) was started with rapid increase of ANC (12,200 cells/μL). Due to the appearance of thrombocytopenia (71 × 10^9^/L) and splenomegaly, treatment with G-CSF was suspended with clinical and laboratory resolution and then re-started at the dose of 4 μg/Kg 3 times/week. At present, the patient is 3 years old, infection-free and his ANC is stably around 2,000 cells/μL. Other than mild and transient splenomegaly, no adverse events have been observed.

## Discussion

Biallelic mutations in the *G6PC3* gene are associated with a variable phenotype. In the two largest series reported until now [4,11] severe neutropenia predisposing to severe infections is the most reliable marker of disease, since congenital heart defects (mostly atrial septal defects) are present in around 80% of patients while prominent superficial veins and facial dysmorphism are frequent but less consistent. Furthermore, six patients with *G6PC3* mutations associated with non syndromic SCN have been described [7]. The role of G6PC3 mutations in causing non hematological manifestations of SCN4 is not yet clear. Neutrophils and skin fibroblasts from SCN4 patients have an increased susceptibility to apoptosis through a mechanism that involves glycogen synthetase kinase 3 beta (GSK-3beta) and endoplasmic reticulum stress. The same mechanism also may play a role in causing dysmorphism [12]. Variable findings have been reported at bone marrow examination: most patients present a maturation arrest at the myelocyte/promyelocyte stage (either in a hypocellurar or hypercellular context), however myelokathexis has been also observed [13]. No genotype-phenotype correlation has been demonstrated to explaine the bone marrow variability as the same mutation can cause either maturation arrest or normo/hypercellular bone marrow [14]. On the contrary, the extra-hematological aspects of the syndrome could be possibly related to the variation in the residual activity of the enzyme, as well as to the genetic or environmental background, since specific missense mutations has been found in association with non-syndromic patients [4,7]. It has been postulated that missense mutations with a higher residual activity could predispose to a non -syndromic phenotype [7]. We herein report on two new cases of SCN4. In addition to contributing two novel *G6PC3* mutations, the patients described also offer some diagnostic and therapeutic observations. Patient 1 illustrates that non-hematopoietic stigmata of the syndrome (including evident facial dysmorphism and vascular anomalies) may appear gradually over time, making the diagnosis difficult early in life. The six patients with non syndromic SCN4 reported were all in the first and second decade of life, the oldest one being 18 years old [7]. Longer follow up studies are needed to assess whether SCN4 is uniformly associated with extra-hematopoietic manifestations (which may also occur later in life) or whether in some patients the clinical phenotype may be restricted to SCN for the entire duration of life. Therefore, *G6PC3* mutation analysis should be considered in all cases of congenital, unexplained neutropenia, regardless of the presence of extra-hematopoietic manifestations or of parental consanguinity, once ELANE and HAX1 mutations are ruled out.

Growth retardation and delayed puberty have been reported in some patients with SCN4 [15]. In one case, hypogonadotrophic hypogonadism was documented, and hypothyroidism was considered an extra-hematological manifestation of SCN4 in another patient [15,16]. Recently, testicular failure with very high levels of luteinizing hormone (LH) and follicle-stimulating hormone (FSH) has been reported in a G6PC3 subject [17]. Patient 1 in this report showed delayed puberty with appearance of irregular menses at 17 years of life. Results of the decapeptyl test were consistent with hypergonadotrophic hypogonadism, and additional tests demonstrated normal estrogen levels and increased LH values, as in partial ovarian insufficiency. The observation of hormonal and growth abnormalities in several patients suggest that appropriate endocrine evaluation should be considered in SCN4 patients.

Sensorineural hearing loss has been previously reported in five patients, all of which were homozygous for a *G6PC3* mutation; moreover parental consanguinity was documented or postulated in several of them [4]. Patient 2 in this report also suffered from sensorineural deafness. Given the relatively high frequency of genetically determined sensorineural deafness in the general population, it remains to be determined whether the latter represents a novel syndromic aspect of the disease, or whether parental consanguinity may have caused simultaneous inheritance of distinct, autosomal recessive genetic defects in *G6PC3* and in another gene responsible for sensorineural deafness.

The *G6PC3* protein is a paralog of G6PC with which it shares 36% sequence identity [3]. Since the *G6PC3* protein structure is not well defined, the functional consequences of the mutations are predicted based on studies performed on the better known G6PC protein. The I125del mutation in patient 1 is located in the transmembrane region and may interfere with the correct folding and stability, in analogy with the effect of missense, transmembrane mutations in the G6PC protein [4]. Patient 2 presents a new complex mutation in exon 6 resulting in amino acid substitution in the endoluminal domain with a premature stop codon after three amino acids. Although no functional studies have been performed to investigate the consequences of this mutation, the occurrence of a premature termination likely interferes with both the structure and the function of the protein.

Recombinant human G-CSF has been successfully employed in the treatment of congenital neutropenia since the end of 80’s , with reduction of the rate of severe infections and improved quality of life, even if some short and long-term adverse effects have been reported [18,19]. Thrombocytopenia and splenomegaly are frequent complications of G-CSF treatment. In patient 1 thrombocytopenia was at first attributed to G-CSF therapy; however, its intermittent course, not related to changes of G-CSF dosage, is similar to what is reported in 10% of *G6PC3* patients, and is therefore most likely related to the disease itself [11]. In patient 2, both splenomegaly and thrombocytopenia were possibly due to the G-CSF treatment, since they regressed after treatment discontinuation, with subsequent reappearence of a mild splenomegaly even at lower doses of G-CSF.

Leukemic transformation is well documented in patients with SCN on G-CSF treatment. Factors contributing to leukemic transformation include the genetic type and severity of neutropenia and G-CSF exposure, i.e. cumulative dose as well as mean dose per injection [20]. Interestingly, no cases of leukemia have been reported in patients with SCN4, possibly due to the young age and the small size of the cohort described [11]. Patient 1 in this report has been treated with G-CSF continuously for 20 years, at a median dose of 3.9 μg/Kg/day (range 2.2-5), with no evidence of leukemia or myelodysplasia on repeated examinations of bone marrow specimens. Nevertheless, a careful evaluation of a possible malignant evolution is warranted.

## Consent

Written informed consent was obtained from the patients for pubblication of this Case report and any accompanying images. A copy of the written consent is available for review by the Editor-in-Chief of this journal.

## Abbreviations

SCN4: Severe Congenital Neutropenia type 4
*G6PC3*: glucose 6 phosphatase catalytic subunit 3
G-CSF: Granulocyte colony-stimulating factor
MDS/AML: Myelodysplasia/acute myeloid leukemia
G6Pase3: Glucose-6-phosphatase 3 protein
GSD1: Glycogen Storage Disease Type 1
DS: Dursun syndrome
PPH: Primary pulmonary hypertension
DHR: Dihydrorhodamine
ANC: Absolute neutrophil count
DHPLC: Denaturing High Pressure Liquid Chromatography
GSK-3beta: Glycogen synthetase kinase 3 beta
LH: Luteinizing hormone
FSH: Follicle-stimulating hormone
